# Quinazoline Derivatives as Targeted Chemotherapeutic Agents

**DOI:** 10.7759/cureus.60662

**Published:** 2024-05-20

**Authors:** Mohamed F Zayed

**Affiliations:** 1 Pharmaceutical Sciences, Fakeeh College for Medical Sciences, Jeddah, SAU

**Keywords:** quinazoline, structural activity relationship, targeted, chemotherapy, cancer

## Abstract

Most of the current chemotherapeutic medications are extremely toxic, exhibit little selectivity, and contribute to the emergence of treatment resistance. Consequently, the discovery of targeted chemotherapy drugs with high selectivity and low side effects is necessary for cancer treatment. The quinazoline system has a broad range and a long history of biological activities. Numerous quinazoline derivatives have been used to treat different types of cancer by working on various molecular targets. This review presents various chemical information, including molecular structure, design, and biological activity of some reported quinazolines that function by inhibiting four types of important molecular targets: dihydrofolate reductase, breast cancer resistant protein, poly-(ADP-ribose)-polymerase, and tubulin polymerization.

## Introduction and background

Cancer is a set of disorders characterized by abnormal cell proliferation and unexplained origins. It possesses the capability of attacking or spreading to other sections of the body, causing many complications that can lead to death [1]. Cancer research accounts for more than 4% of global research. This field of study expands year after year, indicating the global significance of this type of research [2]. Cancer treatment presents numerous challenges due to the variety of complex illnesses. An anticancer medication can be used alone or in conjunction with other medicines. Research into molecular targets and cellular proliferation strategies has resulted in the improvement of numerous anticancer medicines.

**Figure 1:**
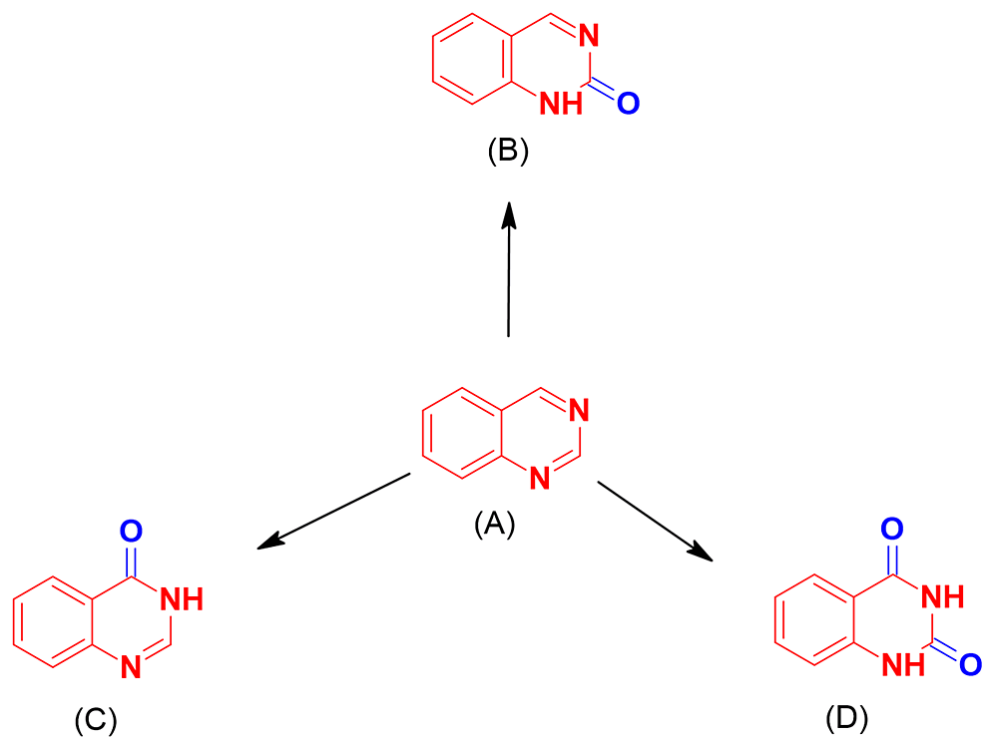
The three structural forms of quinazolines The figure was created by the author using the ChemSketch program.

Previous anticancer agents like alkylating nitrogen mustard molecules, which damage DNA by forming an irreversible covalent bond with the alkyl groups, such as cyclophosphamide and chlorambucil, and antimetabolites, which inhibit DNA synthesis by inhibiting the use of purine metabolites inside the body, such as fluorouracil and mercaptopurine, were employed to treat several forms of cancer. However, they were highly hazardous [3]. Heterocyclic compounds containing nitrogen atoms, such as quinoxaline, phthalazine, and quinazoline, have long been known for their versatile medicinal properties. The quinazoline molecule is a heterocyclic system of phenyl pyrimidine. There are three structural forms of quinazoline as shown in Figure 1 [4].

**Figure 2:**
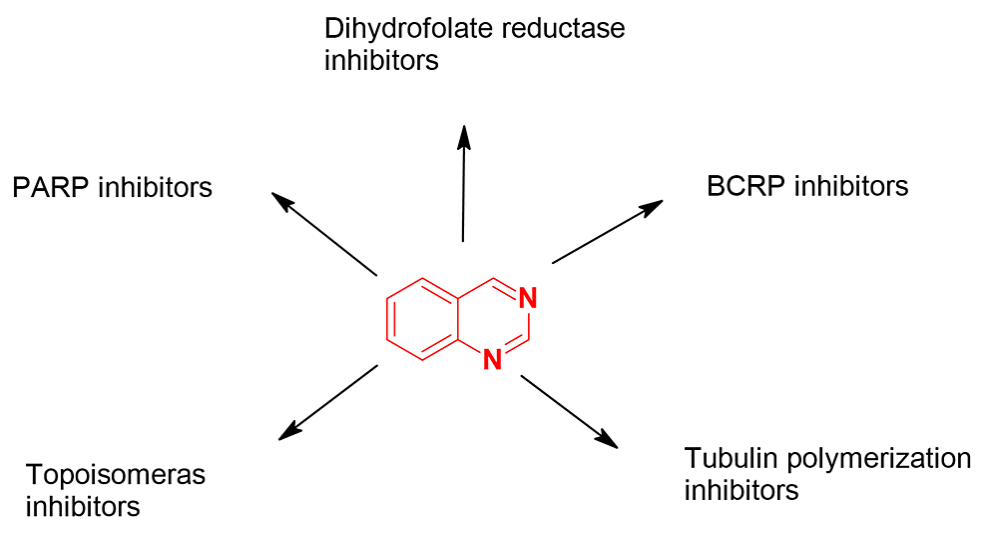
Modes of action of quinazolines The figure was created by the author using the ChemSketch program. PARP: poly(ADP-ribose) polymerase, BCRP: breast cancer-resistant protein.

Quinazolines offer numerous pharmacological properties, including anticancer, antibacterial, antiviral, and many other uses. The reported anticancer quinazolines work by inhibiting different types of enzymes and biological processes [5]. This review assembles some literature about quinazoline molecules that function as targeted chemotherapeutic agents against four important anticancer targets: dihydrofolate reductase (DHFR), breast cancer-resistant protein (BCRP), topoisomerase, poly(ADP-ribose) polymerase (PARP), and tubulin polymerization. Figure 2 shows the different modes of action of anticancer quinazolines, which are discussed in this review article [6].

## Review

Methodology

The search was performed systemically using PubMed, Google Scholar, and ScienceDirect. This search was based on specific keywords, inclusion, and exclusion criteria following Preferred Reporting Items for Systematic Reviews and Meta-Analyses (PRISMA) guidelines.

DHFR inhibitors (DHFRI) 

**Figure 3:**
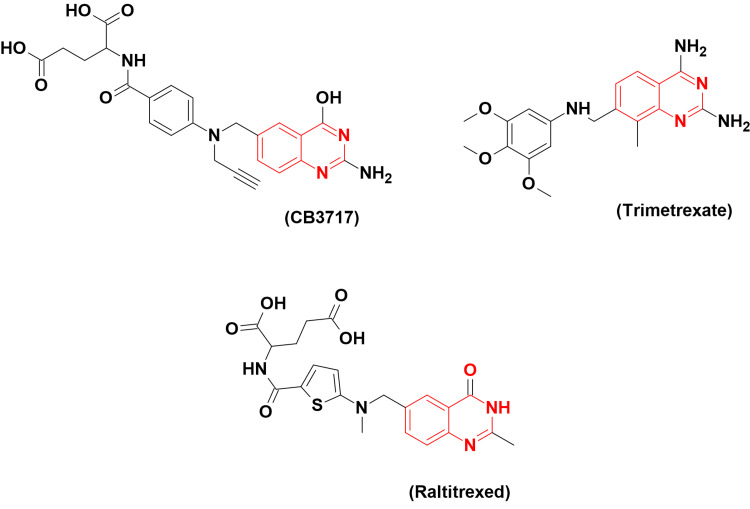
The anticancer CB3717, trimetrexate, and raltitrexed. The figure was created by the author using the ChemSketch program.

The folate analogues are classified as the antimetabolite group among the anticancer agents because they inhibit the metabolic processes required for protein synthesis. The DHFR enzyme is the first well-known target for folate inhibitors. This enzyme converts dihydrofolate to tetrahydrofolate, and then tetrahydrofolate interacts with thymidylate synthase (TS) to contribute to DNA synthesis. TS levels are rising in common kinds of cancer such as breast, colon, and lung cancer. The DHFRI indirectly inhibit TS, which impairs nucleotide biosynthesis [7]. Consequently, DHFR is an important target in cancer chemotherapy. Many quinazoline derivatives work by blocking the action of the DHFR enzyme. CB3717 (Figure 3) was the first quinazoline-based antifolate. After clinical trials, it caused nephrotoxicity and other serious effects. Additionally, it had limited solubility, which led to ceasing these clinical trials for CB3717 as an anticancer agent. Trimetrexate (Figure 3) is another quinazoline-based DHFRI. It had FDA approval for the treatment of leiomyosarcoma. Additionally, it is effective in treating colon cancer and in immunocompromised patients. It is a potent DHFRI with an ID_50_ of 4.75 nM. A dose of 0.1 mM for 24 hours inhibits cell growth by 50-60% of NCI-H630 cell lines [8]. Raltitrexed (Figure 3) is a CB3717 analogue, water-soluble, and does not cause nephrotoxicity due to enhanced water solubility. It had FDA approval for the treatment of colorectal carcinoma and immunomodulating activity with an effective potency (ID_50_ = 52 nM) [9].

**Figure 4:**
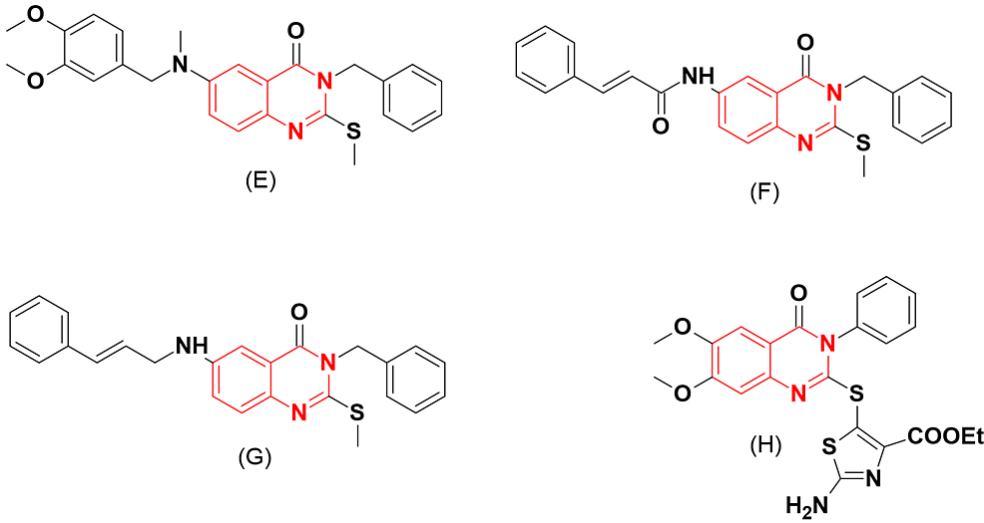
The anticancer derivatives E, F, G, and H. The figure was created by the author using the ChemSketch program.

Al-Rashood and coworkers prepared new molecules of 2,3,4-trisubstituted-quinazoline (Figure 4) and inspected their DHFR inhibitory activity [10]. The quinazoline derivative (E) was the most active one with an IC_50_ = 0.4 μM against an in vitro mammalian cancer cell line. The binding mode of this molecule was similar to that of methotrexate. However, this derivative stopped at the experimental phase and did not proceed to preclinical or clinical studies.

Al-Omary et al. [11] designed other derivatives of quinazoline-based DHFRI (Figure 4). The synthetic approach for these derivatives involved fixing the 3-position with a benzyl group and placing amino (NH_2_) or nitro (NO_2_) fragments at the 6-position, in addition to a cinnamyl thioether or an alkyl group at the 2-position of quinazoline. Among these derivatives, (F) and (G) inhibited the enzyme and showed potent IC_50_ values of 0.6 and 0.5 μM, respectively. Compound (F) showed twice the activity of the reference medication 5-FU in anticancer testing against nine cancer cell lines. The modeling work demonstrated that quinazoline binds to the crucial amino acids Ser59 and Glu30 in the DHFR binding region. These derivatives also did not proceed to further preclinical or clinical studies.

The same research group synthesized 2,6-disubstituted-quinazoline-4-one analogues (Figure 4) based on the results obtained from their previous study [12]. The molecule (H) showed a strong potency with an IC_50_ = 0.3 μM. Structural studies elucidated the importance of the substitutions at the 2-, 3-, and 6-positions and how they influence biological activity. The compounds having a thiazole group at the 2-position were more active than those having nicotinic or nitrobenzoic acid substituents. Docking studies showed the interaction of these derivatives with the amino acids Arg38 and Lys31.

Structure-activity relationship (SAR) of DHFRI

A quinazolin-4-one scaffold with an electron-releasing substituent at the 2-position is required for activity. Changing the 2-, 3-, and 6-substituents resulted in varying the inhibitory activity. Placing a thioalkyl fragment at the C-2 position of quinazoline increased the activity.

BCRP inhibitors

The BCRP is an essential efflux transporter protein that alters and monitors the pharmacokinetic characteristics of several chemotherapeutic agents. It causes anticancer resistance by expelling the anticancer drug from the cancer cell. The physiological function of BCRP is the elimination of toxic materials from the body through the gastrointestinal tract, biliary tract, and placental barrier [13]. Inhibition of BCRP leads to the accumulation of the drug inside the cancerous cell, allowing it to elicit its pharmacological action. The problem of multi-drug resistance (MDR) is treated by co-administration of an anticancer agent with a BCRP inhibitor [14].

**Figure 5:**
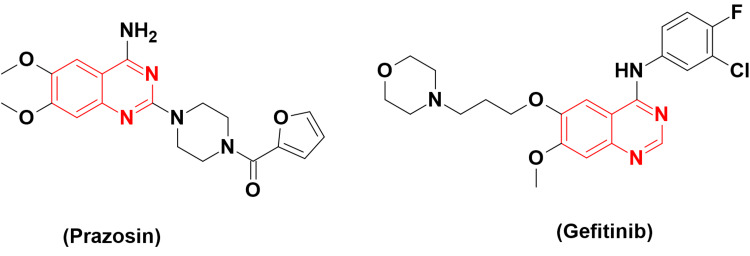
The anticancer prazosin and gefitinib. The figure was created by the author using the ChemSketch program.

Some quinazolines were reported as BCRP inhibitors. Among this class is prazosin (Figure 5), a quinazoline-based BCRP inhibitor molecule. Yanase et al. reported that gefitinib (Figure 5), which works as a tyrosine kinase (TK) inhibitor, also has inhibitory activity against A431 BCRP cells with an IC_50_ of 75.8 nM [15].

**Figure 6:**
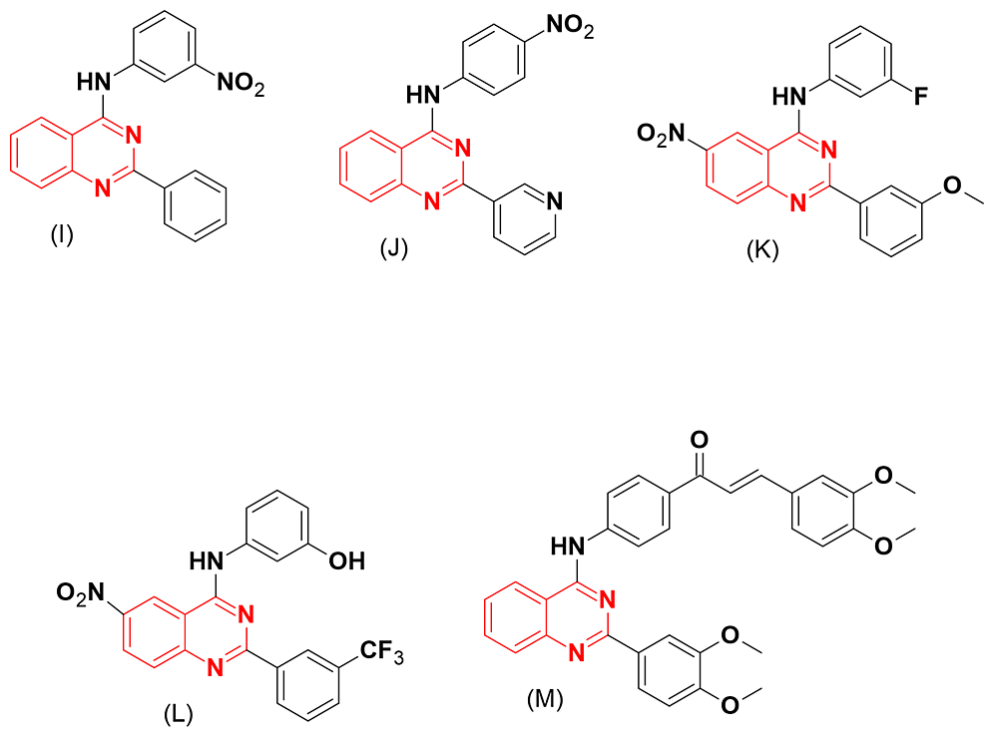
The anticancer derivatives I, J, K, L, and M. The figure was created by the author using the ChemSketch program.

Continuing in this direction, Juval and Wies [16] prepared two series of 2-phenyl-4-anilino-substituted-quinazolines (Figure 6) and tested them for their inhibitory activity of BCRP. Out of these molecules, compound (I), which has a meta-nitro-anilino group, showed potent activity (IC_50_ = 0.13 μM). Further study by the same group revealed that the BCRP inhibitory activity is influenced by 2-, 4-, 6-, and 7-substituents. The phenyl group at the C-2 position of quinazoline is an essential requirement for BCRP inhibition. The meta substituents in the aniline group, such as nitro, hydroxyl, trifluoromethyl, or cyano, are more suitable than the ortho substituents. The dimethoxy-phenyl-substituted-quinazoline analogue of compound (I) displayed potent inhibition (IC_50_ = 0.076 μM).

Considering the previous results, some derivatives of the 2-pyridyl-4-substituted-quinazoline were designed with meta and para substitutions only. The SAR studies showed that both meta and para substituents of the aniline ring at the 4-position were preferred for BCRP inhibitory activity. It also demonstrated that the meta and para pyridyl rings at the C-2 position of quinazoline produced promising activity. Compound (J) displayed an IC_50_ of 64 nM [17].

Based on the previous findings, compounds (K) and (L) displayed strong BCRP inhibitory activity with IC_50_ values of 23.4 and 27.6 nM, respectively, when tested against the ABCG2 cancer cell line. Furthermore, these compounds were able to reverse multidrug resistance and restore sensitivity toward SN-38 and Mitoxantrone [18].

Kraege et al. [19] prepared novel molecules of chalcone-quinazoline hybrids (Figure 6) to test their BCRP inhibitory activity using the MDCK II BCRP cell line. The analogue (M) displayed strong inhibitory activity with an IC_50_ of 0.19 μM.

SAR of BCRP inhibitors

The 4-anilinoquinazoline moiety is essential for activity. The presence of an aniline containing electron-withdrawing groups at the meta or para position increases the activity. The meta-substituted aromatic ring at the C-2 position of the quinazoline ring is preferred for activity. Placing a nitro group at the C-6 position of the quinazoline ring increases the activity.

PARP inhibitors

PARP identifies single-strand DNA breaks (SSBs) caused by chemical or metabolic changes and produces a cellular response to repair the SSBs. There are two types of PARP: PARP-1 and PARP-2. The stimulation of PARP leads to exhaustion of NAD and ADP, negatively affecting mitochondrial functions and leading to cell death [20]. Therefore, PARP inhibition is a target for anticancer agents to program cell death and maintain DNA integrity. Quinazoline-4-one derivatives have displayed an essential role as useful PARP inhibitors.

**Figure 7:**
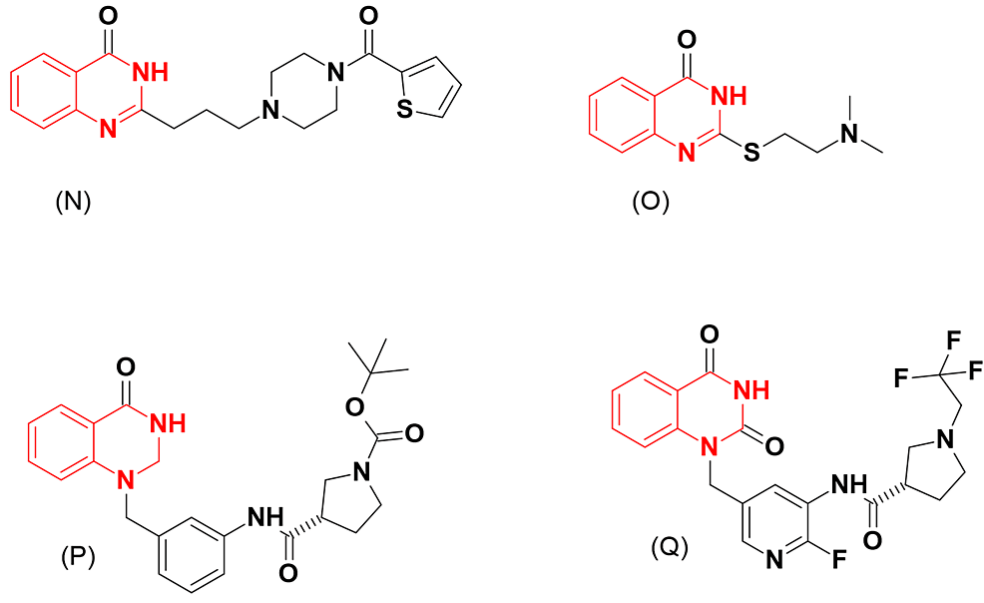
The anticancer derivatives N, O, P, and Q. The figure was created by the author using the ChemSketch program.

Giannini et al. [21] prepared a group of 2-substituted-quinazolinones with the structural requirements of PARP inhibitors (Figure 7). Among these quinazolines, compound (N) produced a high affinity for the inhibition of PARP-1 in mutated carcinoma.

Kulcsar et al. [22] prepared and evaluated a series of 2-substituted-quinazoline-4-ones as PARP inhibitors (Figure 7). Among these newly synthesized compounds, quinazoline (O), having a tertiary amine linked through an S-ethyl linker at the C-2 position of quinazoline, exhibited potent PARP inhibitory activity. Another group of quinazoline-2,4-dione was prepared by Yao et al. [23] to be explored as PARP inhibitors. Quinazoline (P), containing B-proline attached to N1 of the quinazoline moiety, displayed strong inhibitory activity. After studying the molecular structure of PARP-1 and PARP-2, the same research group designed and prepared a new isoform with a terminal pyrrolidine ring (Q). This form showed high selectivity against PARP-1.

**Figure 8:**
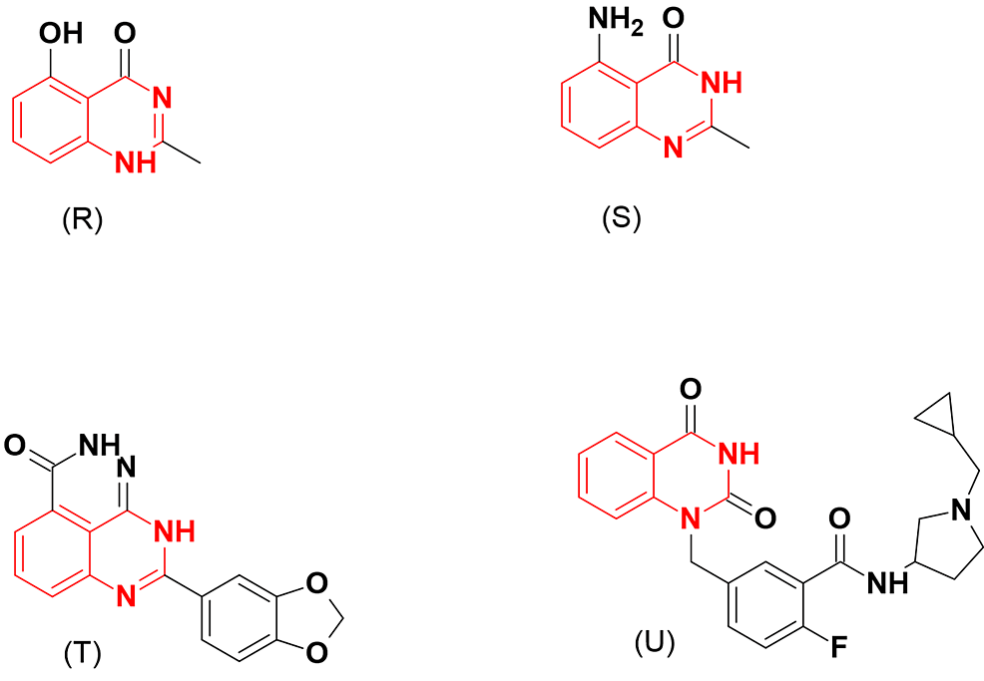
The anticancer derivatives R, S, T, and U. The figure was created by the author using the ChemSketch program.

A group of 2-alkyl/aryl-8-substituted-quinazoline-4-ones was prepared as PARP inhibitors (Figure 8) [24]. Out of this group, compound (R) exhibited significant cytotoxicity in L1210 murine leukemia cells. Kulkarni et al. [25] replaced the hydroxy group of compound (R) with an amino group to form compound (S) to promote aqueous solubility and measure inhibitory activity. They found that compound (S) displayed potent selectivity and cytotoxicity against PARP-1 with an IC_50_ of 49 μM.

In the evaluation of PARP inhibition, the same research group designed a novel class of pyridazino-quinazolin-3-ones. The designed derivatives showed strong inhibitory activity (IC_50_ around 1 μM). SAR studies revealed that electron-donating groups increase activity more than electron-withdrawing groups. Compound (T) in this class showed 100% inhibition of PARP with an IC_50_ of 0.0914 μM.

Further studies explained that small alkyl or aromatic groups at the N1 position of quinazoline were more tolerated than bulky groups. Compound (U) showed strong inhibitory activity with IC_50_ values of 13.3 nM and 67.8 nM against PARP-1 and PARP-2, respectively. Additionally, potent cytotoxic activity against the MX-1 cell line (IC_50_ = 3.2 μM) was obtained [26].

SAR of PARP inhibitors

A quinazoline having a 4-one or 2,4-dione scaffold is required for PARP inhibition. The aromatic ring of the quinazoline system can be substituted at the 5-position with an electron-releasing group or an electron-withdrawing heteroaromatic substituent. Small alkyl or aromatic groups at the N1 position of quinazoline are more favored than bulky substituents. The C-2 position of quinazoline is best joined with electron-donating groups, which are more favorable than electron-withdrawing groups.

Topoisomerase inhibitors

Topoisomerases catalyze the synthesis of DNA by promoting breaking and ligation of the phosphodiester chain in the DNA strands. Inhibition of these enzymes leads to blocking and disruption of the DNA structure [27]. Two types of topoisomerases are identified, I and II, based on whether they work on single or double strands [28]. The regular cell cycle utilizes these enzymes for performing essential processes like transcription, replication, and recombination during DNA synthesis. Consequently, these enzymes are important targets for anticancer agents [29].

**Figure 9:**
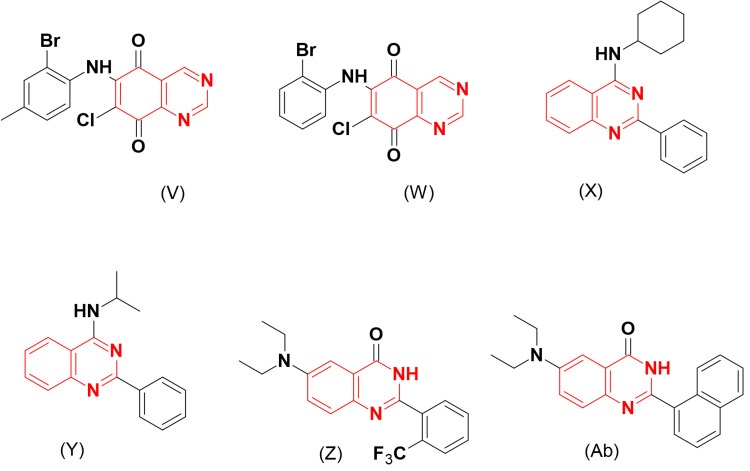
The anticancer derivatives V, W, X, Y, Z, and Ab. The figure was created by the author using the ChemSketch program.

Many quinazoline derivatives were designed as topoisomerase inhibitors. Among them, Park et al. [27] designed 6,7-disubstituted-quinazolin-5,8-dione molecules (Figure 9) for evaluation as topoisomerase inhibitors against lung (A549), stomach (SNU-638), and colon (Col2) cancer cell lines. Bromo, methyl, and methoxy substitutions at the C-2 of an arylamino group attached to the C-6 of quinazoline increased the activity, while substitution at the C-4 decreased the activity. Compounds (V) and (W) displayed strong inhibition on both topoisomerase I and II. The cyclohexylamino-substituted quinazoline (X) and isopropylamino-substituted quinazoline (Y) were evaluated for their cytotoxic activity against different cancer cell lines. They showed potent inhibitions ranging from 2.76 to 16.47 μM. They were also evaluated for their activity against topoisomerase I by means of the supercoiled DNA method and showed potent inhibitions compared to the reference Camptothecin. The SAR study showed that the presence of a phenyl ring at the C-2 of quinazoline plays a vital role in the formation of hydrogen bonds with the enzyme, thereby improving interaction and binding processes [30].

The same group prepared a new series of 2-aryl-substituted quinazoline molecules based on the findings in the previous experiment. The 6-diethylamino-substituted quinazoline derivatives (Z) and (Ab) displayed strong cytotoxic activities against colorectal adenocarcinoma (HCT-15, IC_50_ = 0.28 and 0.1 μM, respectively) and the cervical cancer cell line (HeLa, IC_50_ = 0.46 and 0.004 μM, respectively). These derivatives were more active against topoisomerase I than II [31].

**Figure 10:**
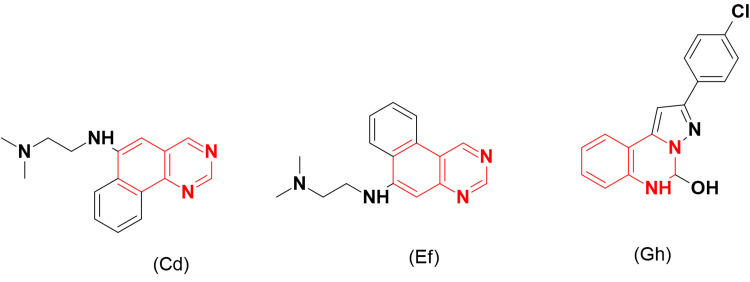
The anticancer derivatives Cd, Ef, and Gh. The figure was created by the author using the ChemSketch program.

Marzaro et al. [32] synthesized another series of benzoquinazolines substituted with a dimethylaminoethyl group (Figure 10) for testing as inhibitors of topoisomerases. The compounds (Cd) and (Ef) showed potent inhibitory activities against A431, HeLa, and HL-60 cancer cell lines, producing IC_50_ values ranging from 3.6 to 23.4 μM. Molecular modeling studies revealed that these compounds have a planar configuration, allowing them to interact with DNA and form a complex.

Kaur et al. [33] designed new derivatives of dihydropyrazolo-substituted-quinazoline and evaluated their cytotoxic activity against topoisomerase II. The compound (Gh) induced considerable activity with an IC_50_ of 3.86 μM, while the standard etoposide had an IC_50_ of 4.32 μM.

SAR of topoisomerase inhibitors

The quinazoline moiety substituted at the C-6 with the 2,4-substituted-arylamino group was more active than the 3-substituted one. Benzoquinazolines with a dimethylaminoethyl group at the 6-position have dual inhibitory activities against topoisomerase I and II. Dihydropyrazolo[1,5-c]quinazolines with an electron-releasing substituent at the 2-position increased the inhibitory activity against topoisomerase II.

Inhibitors of tubulin polymerization

The tubulin protein is composed of assemblies forming microtubules, which display polymerization and depolymerization during cellular division. Drugs targeting microtubules can inhibit or stimulate polymerization, resulting in the destabilization or stabilization of microtubules. Vinca alkaloids and colchicine are examples of naturally occurring tubulin polymerization inhibitors [34]. Some anticancer drugs inhibit tubulin polymerization and cell division, leading to the arrest of the cell cycle at the G2-M phase. Several studies have been conducted to discover and develop quinazoline tubulin polymerization inhibitors.

**Figure 11:**
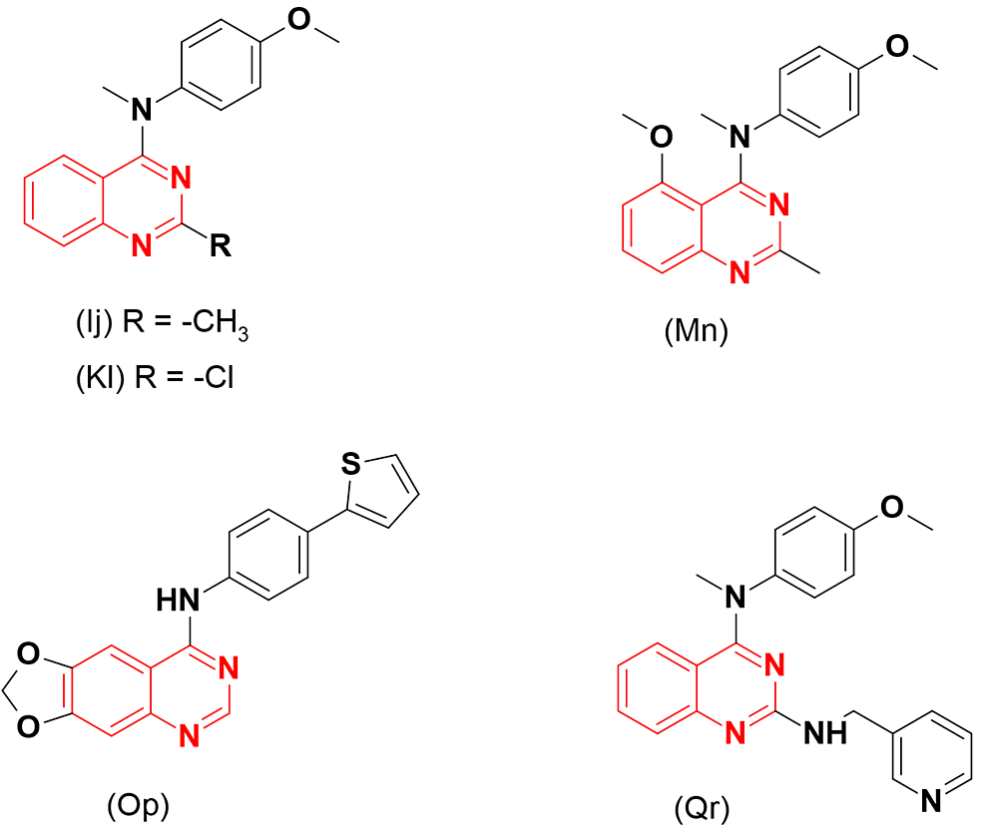
The anticancer derivatives Ij, Kl, Mn, Op, and Qr. The figure was created by the author using the ChemSketch program.

Kasibhatla et al. [35] designed a quinazoline derivative (Figure 11) as a tubulin polymerization inhibitor lead compound, MPC-6827 (Ij), which was named Verbulin. This compound disrupted the mitotic division process and blocked cells at the G2-M phase. However, this compound caused circulatory disorders in phases I and II, leading to the termination of the studies. The same group developed this molecule further by changing the methyl fragment at the C-2 position of quinazoline to a chloro group to investigate its biological activity. The caspase assay showed that the latter compound (MPC-6827, Kl) displayed potent apoptosis with an EC50 of 2 nM against the two cell lines T47D and HCT-116. The inhibitory activity of this molecule was higher than the reference colchicine, with an IC_50_ of 400 nM compared to colchicine's IC_50_ of 500 nM [35]. Additional development of these derivatives by exploring substitutions at the 5-, 6-, and 7-positions of quinazoline produced a new derivative (Mn) with significant tubulin polymerization inhibitory activity [36].

Based on these findings, Marzaro et al. [37] synthesized 4-phenylamino-substituted quinazolines and tested them against different tyrosine kinases. All the derivatives were inactive, while the thiophene derivative (Op) showed strong tubulin polymerization inhibitory activity with an IC_50_ of 1.2 μM. The reference combretastatin A-4 showed an IC_50_ of 1.1 μM. This compound also showed potent cytotoxic activity against several different cell lines.

Li et al. [38] designed some derivatives of 4-anilino-2-substituted quinazoline to inhibit tubulin polymerization. From the designed derivatives, compound (Qr) presented considerable antiproliferative activity (IC_50_ = 0.11 μM) with potent tubulin inhibitory activity (IC_50_ = 2.45 μM).

SAR of tubulin polymerization inhibitors

A quinazoline moiety substituted at the C-4 position with an aminophenyl group is required for tubulin polymerization inhibition. Small lipophilic groups at the C-2 position of quinazoline may increase the activity. An electron-releasing fragment at the C-5 and/or C-6 positions of quinazoline enhances the activity.

Future outlook

Many anticancer quinazolines have been approved by the FDA and are available in the market for clinical use in the treatment of cancer, such as raltitrexed and trimetrexate, which work by inhibiting DHFR. There are also many quinazolines that work by inhibiting tyrosine kinase enzymes, such as gefitinib, erlotinib, vandetanib, dacomitinib, afatinib, canertinib, and lapatinib [39]. These derivatives exhibit some degree of selectivity but still have many side effects, including gastric, hematological, and neurological disorders. Consequently, many other anticancer quinazolines are under experimental or clinical investigation in the hope of producing anticancer agents with greater selectivity and fewer side effects. Third and fourth-generation tyrosine kinase inhibitors are under investigation for future outlook.

## Conclusions

The anticancer quinazolines are a vast group of derivatives that work by inhibiting several molecular targets, such as tyrosine kinase receptors including epidermal growth factor receptor (EGFR), vascular EGFR (VEGFR), and many other targets. However, the present review highlights the use of targeted anticancer quinazolines through the inhibition of DHFR, BCRP, topoisomerase, PARP, and tubulin polymerization. These quinazoline derivatives were primarily designed by modifying the substituents at the C-4, C-5, and C-6 positions of the quinazoline. These small molecular changes led to significant improvements in activity, selectivity, and toxicity. The aforementioned literature survey presented some reported targeted chemotherapeutic quinazolines, their modes of action, and their SAR. It provided a panoramic view for scientists and chemists, inspiring the discovery of new quinazolines as targeted chemotherapeutic agents.
